# Uroflowmetry Parameters in Patients Undergoing Artificial Urinary Sphincter Implantation

**DOI:** 10.1155/aiu/9995075

**Published:** 2025-08-18

**Authors:** Hisanori Taniguchi, Sho Kiyota, Nae Takizawa, Hidefumi Kinoshita

**Affiliations:** Department of Urology and Andrology, Kansai Medical University, Hirakata, Osaka, Japan

**Keywords:** artificial urinary sphincter, incontinence, stress urinary incontinence, uroflowmetry

## Abstract

**Objectives:** The study aims to determine the uroflowmetry parameters of patients undergoing artificial urinary sphincter (AUS) implantation. Hence, uroflowmetry results pre- and post-AUS implantation and differences according to patient background were evaluated.

**Methods:**Thirty-five patients who underwent primary AUS implantation for severe stress urinary incontinence due to radical prostatectomy were enrolled. All patients underwent uroflowmetry tests before and 1, 3, 6, and 12 months after AUS device activation. The patients reported outcomes using validated questionnaires: the King's Health Questionnaire (KHQ), the International Prostate Symptom Score (IPSS), and the quality of life (QOL) score.

**Results:** The mean patient age was 72.8 ± 5.4 years. The mean maximum flow rate (*Q*_max_) value pre-AUS implantation (20.4 ± 11.3 mL/s) was significantly higher at 1-month post-AUS implantation and maintained at 12 months (26.0 ± 14.7 mL/s; *p*=0.011). KHQ, IPSS, and QOL scores were significantly improved after AUS implantation. *Q*_max_, and voiding volume was significantly higher in patients aged < 73 years compared to those aged ≥ 73 years.

**Conclusion:** Uroflowmetry parameters were improved after AUS implantation and maintained for at least 12 months. Not only subjective outcomes but also objective outcomes of uroflowmetry parameters significantly improved after AUS implantation. This is the first report assessing uroflowmetry outcomes after AUS implantation.

## 1. Introduction

For patients with severe stress incontinence, the implantation of an artificial urinary sphincter (AUS) is considered a leading treatment option [1, 2]. Since we established the male incontinence outpatient clinic in 2019, we have been actively implanting AUS [3]. After device activation, an uroflowmetry test is routinely performed to confirm whether patients have no issues with the independent operation of the AUS devices, serving as a useful tool. However, only a few reports have addressed the interpretation of pre- and postoperative uroflowmetry test results in patients undergoing AUS. Han et al. reported that even patients with detrusor underactivity (DU) showed improvements in the mean maximum flow rate (*Q*_max_), postvoid residual urine volume (PVR), and the International Prostate Symptom Score (IPSS) after AUS implantation [4]. Similarly, Kataoka et al. indicated that patients with a history of pelvic radiation therapy showed initial improvements but tended to experience deterioration in lower urinary tract symptoms over time [5]. Therefore, the study aimed to determine the uroflowmetry parameters of patients undergoing AUS implantation. Furthermore, this study investigated the uroflowmetry results pre- and post-AUS implantation and differences according to patient background.

## 2. Materials and Methods

This retrospective study was approved by the ethics committee of our institution (approval no. 2018086). All participants provided written informed consent.

We included 35 out of 50 patients who underwent single-surgeon primary AUS implantation for severe stress urinary incontinence due to radical prostatectomy between March 2019 and October 2023 at our institution and followed up for at least 12 months after device activation. Eight patients who did not undergo uroflowmetry before treatment, 6 patients with inadequate questionnaire responses, and 1 patient whose AUS were removed due to cognitive progression after AUS implantation were excluded from this study. Flexible cystoscopy was routinely performed to check for a urethral stricture as a standard pre-AUS implantation examination. Although a pressure-flow study was performed in some patients as part of the preoperative evaluation, the data were not available for all participants and therefore were not included in the present analysis. Instead, this study focused on noninvasive assessments, including uroflowmetry and cystometry, to evaluate lower urinary tract function.

All AUS implantations were conducted using AMS800® (Boston Scientific, Marlborough, MA, USA) following a standard procedure by a perineal approach. All patients received urethral cuffs measuring either 4.0 or 4.5 cm. The reservoir, filled with 22 mL of normal saline, was positioned in the perivesical retroperitoneal space via an additional supra-inguinal incision. The control pump was implanted in the right scrotum through the same incision. [6]. The pump was deactivated at the end of the surgery, with devices activated 6 weeks after AUS implantation.

All patients underwent uroflowmetry tests before and 1, 3, 6, and 12 months after AUS device activation. Pre-AUS implantation urodynamic data were obtained to confirm unobstructed status. We measured the cystometric capacity for filling with saline in a supine position, followed by the uroflowmetry test in the standing position. A post-AUS implantation uroflowmetry test was performed, asking patients to urinate as much volume as possible.

The patients reported outcomes using validated questionnaires, such as the King's Health Questionnaire (KHQ) [7], IPSS [8], and quality of life (QOL) score, at pre- and post-AUS implantation outpatient visits. Additionally, the patient's self-reported continence status of the number of daily pad uses was evaluated. Patients' characteristics and clinical outcomes were investigated using both subjective and objective assessment tools pre- and post-AUS implantation. The differences in the uroflowmetry test according to patients' characteristics were also examined.

### 2.1. Statistical Analysis

All statistical analyses were performed using SPSS Statistics version 28.0.1 (IBM Corp., Armonk, NY, USA). Continuous data are presented as a mean ± standard deviation (SD) and number (percentage). In all figures, error bars represent the standard error (SE) of the mean, unless otherwise indicated. Comparison between pre- and post-AUS outpatient visiting data in the two groups was performed using the paired *t*-test and Mann–Whitney *U*-test, respectively. A *p* value < 0.05 indicated statistical significance. This study was conducted in accordance with the Helsinki Declaration and ethical guidelines for clinical research.

## 3. Results

### 3.1. Patient Background

Table 1 demonstrates patients' pre-AUS implantation characteristics. The mean patient age was 72.8 ± 5.4 years, while the mean duration from the initial cause of stress incontinence to AUS implantation was 68.9 ± 56.1 months. Nine (25.7%) patients were diagnosed with diabetes mellitus (DM) preimplantation and treated with medications.

### 3.2. Subjective Outcomes Assessed by Questionnaires

**Figure 1:**
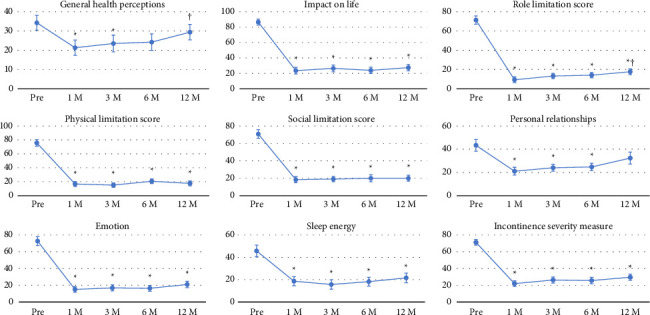
Changes in the urinary-related quality of life mean scores assessed using the King's Health Questionnaire. Data are presented as mean ± standard error (SE). The *x*-axis shows the time points (Pre, 1, 3, 6, and 12 months after device activation), and the *y*-axis indicates the scores. ^∗^*p* < 0.05 versus Pre, ^†^*p* < 0.05 versus 1 month after AUS activation.

**Figure 2:**
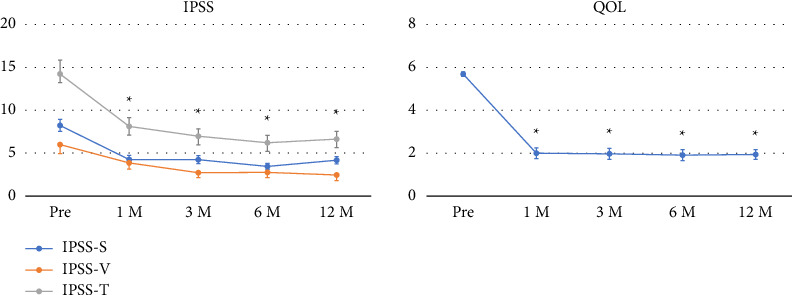
Changes in the International Prostate Symptom Score (IPSS) and quality of life (QOL) scores after AUS activation. Data are presented as mean ± standard error (SE). The *x*-axis shows the time points (Pre, 1, 3, 6, and 12 months after device activation). The *y*-axis indicates the scores of the following domains: IPSS-S, storage subscore; IPSS-V, voiding subscore; IPSS-T, total score; QOL, quality of life. ^∗^*p* < 0.05 versus Pre.

Figure 1 presents patient-reported outcomes using the KHQ score, whereas Figure 2 demonstrates patients' IPSS and QOL scores. All KHQ items were significantly improved 1 month after AUS implantation and maintained at 12 months except for general health perceptions and personal relationship subscores (Figure 1). Additionally, IPSS and QOL scores were significantly improved 1 month after AUS implantation and maintained at 12 months (Figure 2).

### 3.3. Objective Outcomes of Daily Pad Use and Uroflowmetry Parameters

**Figure 3:**
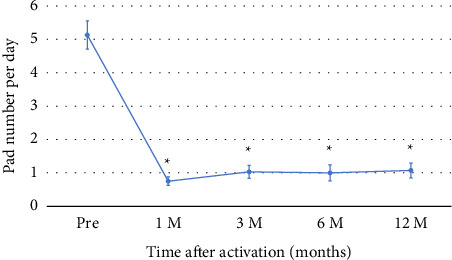
Changes in the mean number of daily pad uses after AUS activation. Data are presented as mean ± standard error (SE). The *x*-axis shows the time points (Pre, 1, 3, 6, and 12 months after device activation), and the *y*-axis indicates the average number of pads used per day. ^∗^*p* < 0.05 versus Pre.

**Figure 4:**
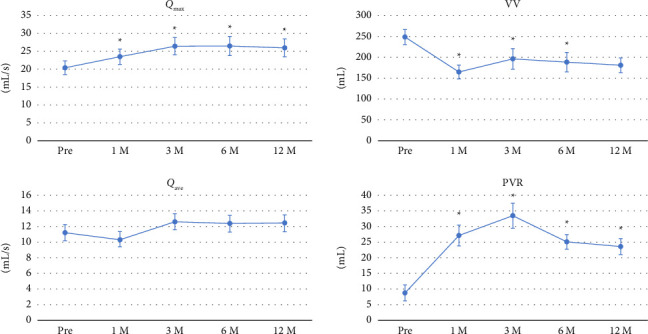
Changes in uroflowmetry parameters before and after artificial urinary sphincter (AUS) implantation. Data are presented as mean ± standard error (SE). The *x*-axis shows the time points (Pre, 1, 3, 6, and 12 months after device activation). The *y*-axis indicates each uroflowmetry parameter: *Q*_max_, maximum flow rate (mL/s); *Q*_ave_, average flow rate (mL/s); VV, voiding volume (mL); PVR, postvoid residual volume (mL). ^∗^*p* < 0.05 versus Pre.

The mean number of daily pads used was significantly decreased from 5.13 ± 2.5 before implantation to 0.75 ± 0.8 at 1 month after AUS implantation and maintained at 12 months at 1.07 ± 1.5 (Figure 3).  Figure 4 shows the changes in uroflowmetry parameters according to AUS implantation. The *Q*_max_ value before AUS implantation was significantly lower than after AUS implantation (20.4 ± 11.3 mL/s before AUS implantation vs. 26.0 ± 14.7 mL/s at 12 months after AUS implantation; *p*=0.011). The average flow rate (*Q*_ave_) and voiding volume (VV) did not significantly change, comparing those before and 12 months after AUS implantation. On the other hand, PVR was significantly increased (8.74 ± 15.2 mL before AUS implantation vs. 23.6 ± 15.0 mL at 12 months after AUS implantation; *p* < 0.001).

### 3.4. Comparisons of 12-Month Postoperative Urinary-Related Outcomes of Different Groups

We categorized patients into two groups according to their age (< or ≥ 73 years), DM diagnosis, and time from the initial cause of stress incontinence to AUS implantation (TT-AUS; < or ≥ 70 months).  Table 2 presents urinary-related outcomes compared between these two groups. Despite no significant difference between the two groups based on DM diagnosis and TT-AUS, *Q*_max_ and VV were significantly higher in patients aged < 73 years compared with patients aged ≥ 73 years.

## 4. Discussion

This retrospective observational study focusing on uroflowmetry parameters of patients undergoing AUS implantation showed that *Q*_max_ values were significantly higher at 1 month after AUS implantation compared with pre-AUS implantation and maintained for at least 12 months. Patient-reported subjective outcomes assessed by KHQ and IPSS questionnaires also showed significant improvement. Additionally, regarding the urinary-related outcomes, *Q*_max_ and VV were significantly higher in patients aged < 73 years compared with patients aged ≥ 73 years at 12 months after implantation. The presence or absence of DM and TT-AUS did not influence the uroflowmetry results.

AUS implantation for severe stress incontinence after radical prostatectomy represents the gold standard treatment option, improving patients' QOL [9]. Accordingly, several studies reported that AUS improved not only stress incontinence but also QOL [9, 10]. However, only a few uroflowmetry outcomes related to AUS implantation have not been explored [4, 5].

This study investigated a population with a mean patient age of 72.8 years and a duration from the initial cause of incontinence to AUS implantation of 68.9 months, showing significantly higher mean maximal flow rate values at 1 month after implantation compared with that before implantation, which were maintained for at least 12 months. All patients underwent radical prostatectomy and mainly without additional radiation therapy (77.1% of all participants). However, the filling phase of the uroflowmetry test was evaluated in the spine position for patients with severe stress incontinence. Furthermore, this study could assess the postoperative course of uroflowmetry parameters over time. We demonstrated not only the improvement of urinary-related subjective QOL but also the improvement in uroflowmetry parameters, which were maintained for at least 12 months post-AUS implantation. These results are consistent with that of Han et al., who reported that DU does not necessarily impair AUS-related outcomes for uroflowmetry and symptom improvement [4]. Moreover, since our cohort comprised mainly patients who had not undergone radiotherapy, we did not observe long-term deterioration in urinary symptoms. This finding contrasts with those by Kataoka et al., which show that LUTS deterioration occurred after AUS implantation in patients with a history of pelvic radiation therapy [5].

Although the uroflowmetry test is a noninvasive test assessing urinary tract dysfunction, its flow rates vary depending on age, gender, VV, and urethral physiology. Furthermore, some studies established a nomogram chart to provide a normal reference range for the urinary flow rate. Shinohara et al. developed a nomogram for urinary volume and flow using data from Japanese men without lower urinary tract symptoms and *Q*_max_ of > 150 mL, which were compared among different age groups. Their study showed that the mean *Q*_max_ in (21.8 ± 5.05 mL/s) was significantly lower in the 50–59 age group compared to aged < 50 years [11]. Kumar et al. demonstrated that the median age of 67 years with a median VV of 297 mL showed *Q*_max_ and *Q*_ave_ of 17 ± 7.6 mL/s and 8.9 ± 4.0 mL/s, respectively, which were well correlated with voided volume. They also showed the negative correlation of *Q*_max_ and *Q*_ave_ with age in this group [12]. Our study demonstrated that the mean *Q*_max_ and *Q*_ave_ at 12 months post-AUS implantation were 26.0 ± 14.7 mL/s and 12.5 ± 6.6 mL/s, respectively. These results were not lower than in previous reports. However, although *Q*_max_ was lower in patients aged ≥ 73 years compared to those under 73, it did not affect subjective QOL scores. Although the *Q*_max_ values were significantly lower in patients aged ≥ 73 years compared with younger patients, subjective QOL or IPSS scores demonstrated no significant differences between the two groups. This apparent discrepancy suggests that the effectiveness of AUS in restoring continence and improving patient-perceived QOL is not solely dependent on urinary flow rate. Thus, older patients may experience a similar degree of satisfaction and symptom relief, possibly due to lower baseline expectations or greater tolerance for mild voiding inefficiency. Furthermore, as long as continence is restored and voiding is functionally adequate, minor reductions in flow rate may not substantially affect the perceived QOL.

In addition to the age-related findings, while both *Q*_max_ and PVR increased after AUS implantation, VV slightly decreased. This pattern may reflect adaptive voiding behavior among patients who choose to void more frequently in smaller amounts to prevent potential leakage or discomfort, especially during the adjustment period early after device activation. Furthermore, the inherent resistance from the artificial sphincter may mildly increase the residual urine volume. Importantly, these physiological changes were not associated with a decline in patient-reported QOL or IPSS scores; therefore, patients functionally adapted to these changes without perceiving them as detrimental.

Uroflowmetry data were not worse in patients with DM compared with patients without DM in this study. Previous studies reported DM as a risk factor for operative complications and revision surgery [13, 14]. However, from the uroflowmetry viewpoint, this study showed that DM is not an inferior factor of AUS implantation. Kaiho et al. also revealed that continence and QOL could be improved in patients with and without DM [15].

This study has a number of limitations. First, it included a small sample size. Second, the study utilized a retrospective nature without long-term follow-up. Nevertheless, to the best of our knowledge, this is the first study evaluating uroflowmetry parameters pre- and post-AUS implantation. Hence, additional adequately powered and prospective studies with well-characterized populations are required to verify our findings. Furthermore, while this study separately analyzed each uroflowmetry parameter (*Q*_max_, *Q*_ave_, VV, and PVR), future research may benefit from developing a composite scoring system that integrates these variables into a single metric. Such a score could offer a more comprehensive and clinically intuitive assessment of voiding function after AUS implantation. Additionally, further prospective studies with larger cohorts are warranted to validate the utility and predictive value of such a scoring approach.

In conclusion, the uroflowmetry parameters of patients undergoing AUS implantation improved after postimplantation and were maintained for at least 12 months post-AUS implantation. These objective findings may contribute to improved subjective evaluations, including QOL.

## Figures and Tables

**Table 1:** Patients' background and uroflow study data pre-AUS implantation.

Variable	Number (%) or mean ± SD
Number of patients	35
Age (year)	72.8 ± 5.4
Body mass index (kg/m^2^)	24.3 ± 3.1
Diabetes mellitus (+)	9 (25.7)
Duration from initial cause of incontinence to AUS (mo.)	68.9 ± 56.1
Cause of incontinence	
RARP	19
LRP	6
ORP	10
Radiation therapy before AUS	8 (22.9)
Number of daily pads use	5.13 ± 2.5

Results of uroflow study	
Maximal flow rate (mL/s)	20.5 ± 11.3
Average flow rate (mL/s)	11.2 ± 6.1
Voiding volume (mL)	248.8 ± 106.7
Postvoid residual urine (mL)	8.7 ± 15.1

IPSS total	14.2 ± 9.5
Voiding subscore	6.0 ± 6.2
Storage subscore	8.2 ± 4.1
QOL score	5.7 ± 0.6

Abbreviations: AUS = artificial urinary sphincter, IPSS = International Prostatic Symptom Score, LRP = laparoscopic radical prostatectomy, ORP = open radical prostatectomy, QOL = quality of life, RARP = robot-assisted radical prostatectomy.

**Table 2:** Comparisons of 12-month postoperative urinary-related outcomes of different groups (*n* = 35).

Variables	Age (years)		*p* value	DM		*p* value	Time to AUS (mo.)		*p* value
	< 73	≧ 73		+	—		< 70	≧ 70	
IPSS total score	6.33 ± 4.8	6.89 ± 6.0	0.77	6.75 ± 6.0	6.33 ± 3.8	0.81	6.57 ± 4.2	6.80 ± 7.8	0.93
QOL	1.73 ± 1.2	2.11 ± 1.5	0.43	1.88 ± 1.2	2.11 ± 1.76	0.71	1.83 ± 1.0	2.20 ± 1.9	0.58
*Q*_max_ (mL/s)	32.0 ± 17.0	20.4 ± 9.8	0.025	28.6 ± 14.2	25.2 ± 15.1	0.31	24.3 ± 13.1	31.4 ± 18.4	0.25
*Q*_ave._ (mL/s)	14.8 ± 7.6	10.3 ± 4.8	0.054	13.4 ± 6.3	12.1 ± 6.8	0.35	11.8 ± 6.1	15.1 ± 8.7	0.27
VV (mL)	227.4 ± 117.5	137.4 ± 65.2	0.013	187.8 ± 100.1	178.9 ± 106.3	0.67	177.4 ± 101.7	204.7 ± 120.2	0.51
PVR (mL)	21.8 ± 16.3	25.3 ± 14.0	0.51	20.3 ± 11.6	24.6 ± 16.1	0.79	25.4 ± 16.8	24.2 ± 17.6	0.85

Note: PVR = postvoid residual urine volume, *Q*_ave._ = average flow rate, *Q*_max._ = maximum flow rate.

Abbreviations: AUS = artificial urinary sphincter, DM = diabetes mellitus, IPSS = International Prostatic Symptom Score, QOL = quality of life, VV = voiding volume.

## Data Availability

The data that support the findings of this study are available on request from the corresponding author. The data are not publicly available due to privacy or ethical restrictions.
